# Use of an Aortic Cannula for Tracheal Intubation in a Patient With Severe Tracheal Stenosis and Tracheoesophageal Fistula: A Case Report

**DOI:** 10.7759/cureus.9456

**Published:** 2020-07-29

**Authors:** Asad Siddiqui, Nikolaus E Wolter, Clyde Matava

**Affiliations:** 1 Anesthesia and Pain Medicine, Hospital for Sick Children, Toronto, CAN; 2 Otolaryngology - Head and Neck Surgery, Hospital for Sick Children, Toronto, CAN; 3 Anesthesiology and Pain Medicine, University of Toronto, Toronto, CAN

**Keywords:** airway, aortic cannula, trachea, fistula, pediatric, congenital, anesthesia, difficult airway, 3d reconstruction

## Abstract

A one-day-old girl was brought to the OR for the repair of a type C esophageal atresia (EA) [EA with tracheoesophageal fistula (TEF)]. Rigid bronchoscopy was performed to locate the fistula, and it revealed a severe long-segment tracheal stenosis. Therefore, the airway could not have been secured past the fistula using normal-sized endotracheal tubes (ETTs). A nontraditional airway using an aortic cannula was used to intubate the stenotic tracheal segment, and the patient received ventilation during the TEF/EA repair.

## Introduction

Complete tracheal rings with concurrent tracheaesophagal fistula (TEF) are extremely rare and pose unique challenges for ventilatory management during the operative repair [1]. A one-day-old girl weighing 2.7 kg presented to the OR for repair of a type C esophageal atresia (EA) with TEF. Following induction of anesthesia, rigid bronchoscopy was performed by otolaryngology, and it was noted that the child had a TEF in the presence of a severe long-segment tracheal stenosis beyond the fistula. The fistula was at the level of the tracheal stenosis with bile spilling into the stenotic entrance. Given the diameter of the stenotic portion of the airway, there would be significant difficulty in isolating beyond the fistula using traditional techniques with normal-sized endotracheal tubes (ETTs). Using a previously published approach identified in an institutional case report, an aortic cannula was used to intubate the airway and provide ventilation in the stenotic portion distal to the fistula during the TEF repair [2]. 

## Case presentation

A one-day-old girl, who was born at term weighing 2.7 kg, required emergency repair of type C EA (EA with TEF). TEF had been diagnosed following vomiting after every feed and failure to pass a nasogastric tube into the stomach based on chest and abdominal radiographs depicted a pouch at the T2-3 level and air in the stomach, respectively. Echocardiography revealed normal cardiac anatomy and function. The patient required 120 mL/min of supplemental oxygen administered via nasal prongs and was still experiencing desaturations because of thick oropharyngeal secretions. A Replogle tube was in situ to drain the secretions. After transferring her to the OR, general anesthesia was induced with a propofol infusion at 600 mcg/kg/min, which gradually titrated down to 400 mcg/kg/min. Subsequently, 1 µg/kg of dexmedetomidine was administered as a bolus over 10 min. Following the induction of anesthesia and during rigid bronchoscopy, the otolaryngologist discovered severe tracheal stenosis with complete tracheal rings. The patient was transferred back to the neonatal intensive care unit (NICU) for contrast-enhanced CT of the neck and chest to rule out the presence of vascular rings and other anatomical abnormalities. She was breathing spontaneously with a Guedel airway and supplemental oxygen via a face mask. The CT demonstrated congenital tracheal stenosis with marked narrowing of the thoracic trachea measuring 1.6 mm × 2.2 mm in the narrowest segment with no vascular rings or slings. The patient was rescheduled for the OR to repair the TEF/EA. Written consent was obtained from the parents to publish this case. 

**Figure 1:**
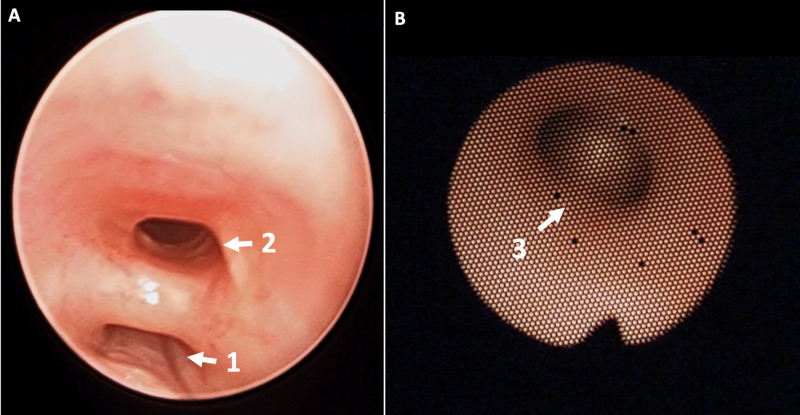
Bronchoscopic findings. A. Rigid bronchoscopy demonstrating the proximal end of the severely stenotic trachea (arrow 1) with a large TEF (arrow 2). B. Flexible bronchoscopy through the stenotic tracheal segment with the carina (arrow 3). TEF, tracheoesophageal fistula

Before returning to the OR, a multi-disciplinary meeting with the anesthesia, otolaryngology, general surgery, and cardiac surgery teams was held to discuss the plan for this rare diagnosis [1]. Table 1 summarizes the major discussion points, concerns, and compromises regarding the case. Figure 1 illustrates the findings during the initial rigid bronchoscope. 

**Table 1:** Major discussion points in the multi-disciplinary meeting. TEF, tracheoesophageal fistula; ECMO, extracorporeal membrane oxygenation; ETTs, endotracheal tubes; EA, esophageal atresia

Specialty	Concerns	Discussions	Final plan
Anesthesiology	Severe tracheal stenosis Risk of soiling the lungs Risk of complete airway obstruction during the procedure with gastric contents and blood	No ETTs available to intubate the stenotic trachea Need to identify a tube/device that allows intubation of the stenotic trachea, while preventing soiling of the trachea, and preferential ventilation through the fistula into the stomach	Plan A: Aortic cannula placement for ventilation Plan B: ECMO
General surgery	Cannot delay TEF repair because of the risk of chronic soiling of the lungs	The concern of respiratory complications from chronic lung soiling Risks of morbidity and mortality due to ECMO in a small child	Thoracotomy to ligate the fistula Compromise: delayed repair of the EA
Otolaryngology	Severe tracheal stenosis, which should not be worsened during the airway manipulation	The stenotic tracheal segment should not be manipulated	In case of elective cannulation with ECMO, full repair with slide tracheoplasty should be performed
Cardiac surgery	Difficult to perform emergency ECMO cannulation in the lateral position on a small child	Consider elective cannulation for ECMO before repair, if necessary	Elective cannulation and de-cannulation of ECMO to allow for repair

 

This severely stenotic tracheal section was immediately distal to the sizeable esophageal fistula and approximately 18 mm distal to the inferior border of the glottic opening (Figure 1 A, B and Video 1).

**Video 1:** Video depicting the first rigid bronchoscopy, aortic cannula bronchoscopy, real-time flexible bronchoscopy, and post-repair rigid bronchoscopy.

**Figure 2:**
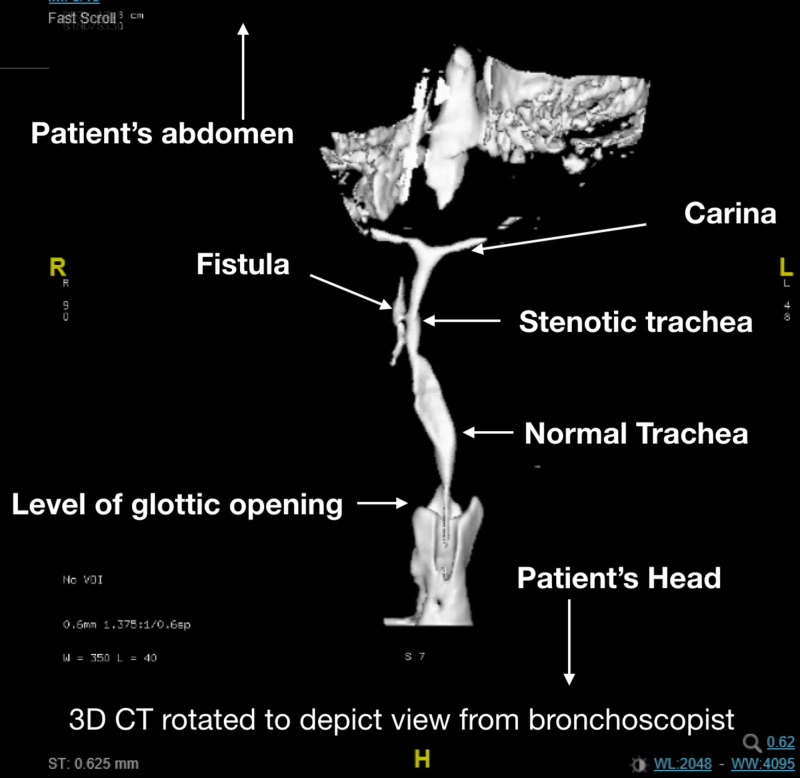
Enhanced 3D reconstruction and subtraction of the trachea. Three-dimensional CT airway reconstruction rotated to depict the anatomy on bronchoscopy in a patient in the supine position.

The anesthesia team reviewed the rigid bronchoscopy video and created a 3D reconstruction of the airway using CT images (Figure 2).

An airway management strategy was developed, which included the use of a tube with an outer diameter of less than 2.2 mm (Figure 2). This strategy had the following goals:

1) secure and maintain airway continuity through the stenotic tracheal segment;

2) prevent soiling of the lungs;

3) minimize the possibility of tube migration and displacement into the fistula during the thoracic surgery;

4) allow positive pressure ventilation; and

5) avoid injury to the tracheal stenosis segment of the airway.

The smallest institutional ETT, with an internal diameter of 2.0 mm and an outer diameter of 2.9 mm, would not fit the stenotic airway segment. 

**Figure 3:**
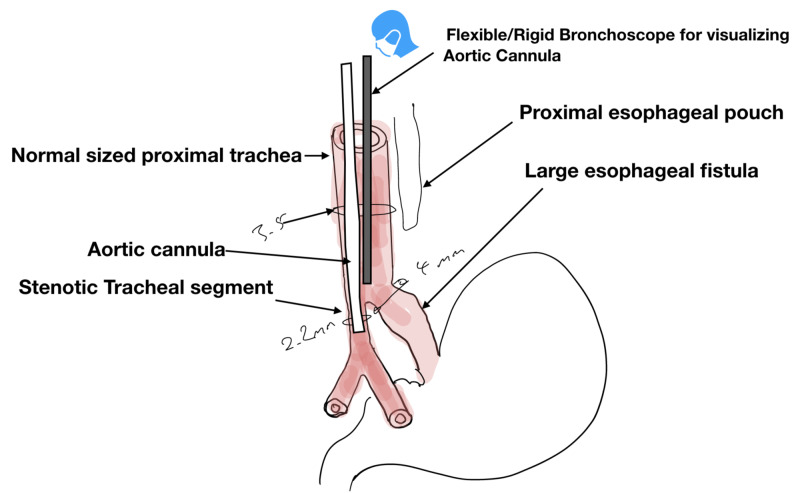
Schematic diagram of the final plan for the airway. Schematic diagram of the final plan for the airway depicting the planned approach to manage the patient

The anesthesia team discussed the options for securing the airway with the colleagues. Peliowski and Holtby (2006) had reported the use of a 6-FR aortic cannula to secure the airway of an eight-month-old patient with tracheal stenosis during emergency cartilage-patch tracheoplasty [2]. However, the present case was different in that a TEF/EA repair and postoperative extubation were required. Therefore, tracheal damage could contribute to increased morbidity and possible mortality in the postoperative phase. The anesthesia team tested and evaluated the 6-FR aortic cannula, which had an outer diameter of 2.0 mm and a soft tip reinforced with small wire coils, making it ideal for wedging or intubating the proximal portion of the tracheal stenosis (Figure 3).

**Figure 4:**
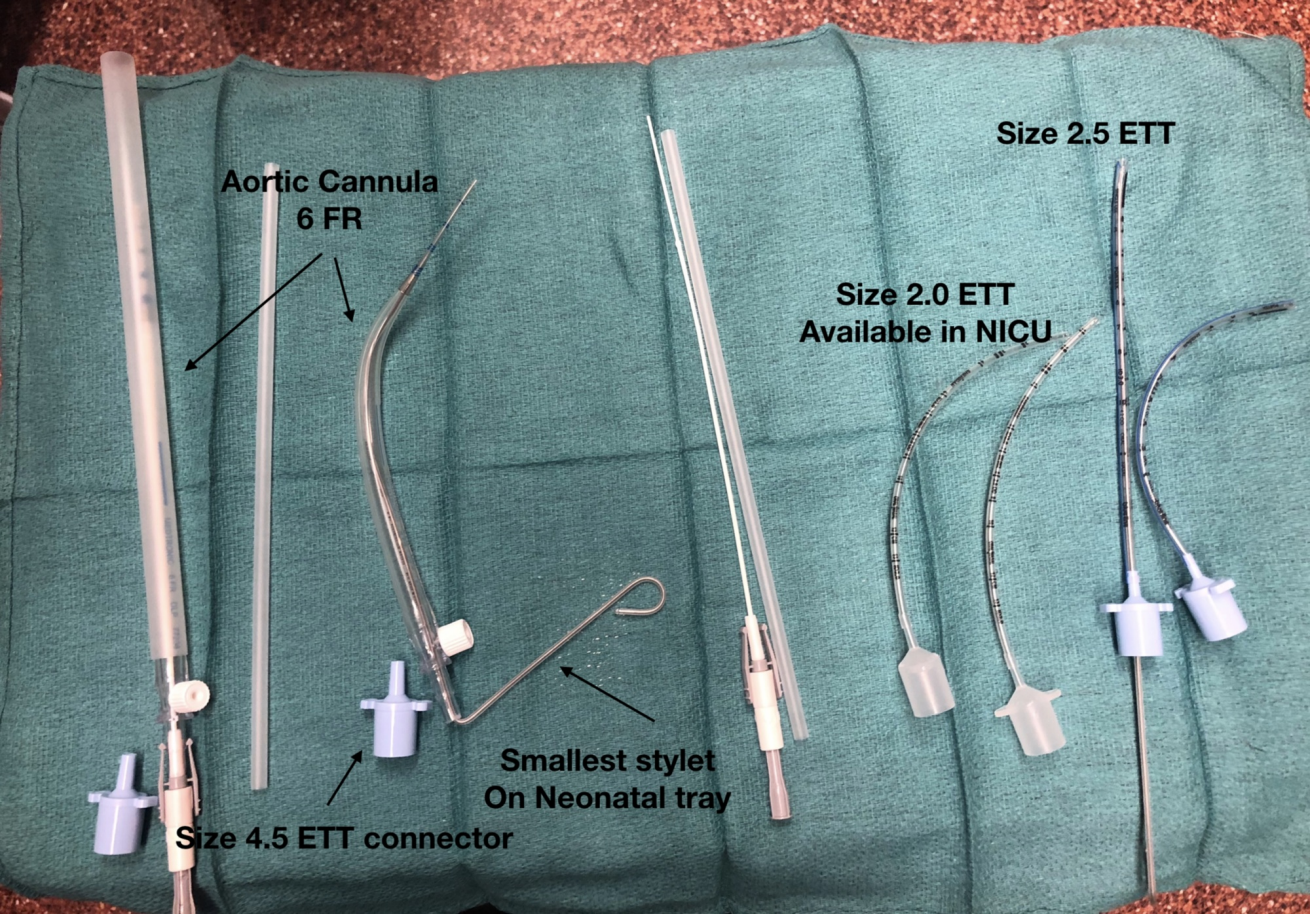
Array of the aortic cannula and ETTs. ETTs, endotracheal tubes

Its proximal end accommodates a 4.5-mm ETT adapter to connect it to the ventilator (Figure 4). Discussions were held with the parents at every stage, and the risks and mitigation plans were reviewed. A back-up plan, which included the initiation of extracorporeal membrane oxygenation (ECMO) in the event of complete respiratory failure was established.

The patient was transported to the OR, and the standard monitors were set-up. General anesthesia was induced with a propofol infusion of 500 µg/kg/min, a bolus infusion containing 1 µg/kg of dexmedetomidine over 10 min, and 5 µg/kg of glycopyrrolate. These were supplemented with intermittent boluses of propofol (total 10 mg/kg) and fentanyl (total 1.5 µg/kg) in the first stage of the procedure. Steroids were administered to prevent mucosal swelling. The goal of the first stage was to secure the airway through the insertion of the aortic cannula in the stenotic tracheal segment, visualized with a flexible bronchoscope (Figure 3). Spontaneous ventilation and adequate depth of anesthesia were maintained throughout the first stage. Our initial plan for airway management included using a flexible bronchoscope and GlideScope™ (Verathon Inc, Bothell, WA) to guide the aortic cannula across the vocal cords. However, the vocal cords could not be visualized with either the video laryngoscope or the flexible scope. After two failed attempts, we used a rigid bronchoscope to visualize the vocal cords and insert the aortic cannula. 

In collaboration with the otolaryngology team and with the use of a rigid bronchoscope, the anesthesiologist guided the aortic cannula into the tracheal stenosis. The rigid bronchoscope was positioned posterior to the aortic cannula to allow continuous visualization of the cannula’s advancement into the stenotic tracheal segment. The correct placement of the aortic cannula would insert 1 cm of the flexible tip in the stenotic tracheal segment, which was confirmed visually. Subsequently, a flexible bronchoscope was inserted orally into the trachea and placed adjacent to the aortic cannula to allow continuous video monitoring (Video 1). This allowed us to visualize and identify any migration of the aortic cannula inside or outside the stenotic tracheal segment. Once the airway was secured, a radial arterial line was placed, and the patient was placed in the left lateral decubitus position. The second stage was the surgical repair of the TEF/EA. General anesthesia was maintained with a combination of propofol and sevoflurane infusions. Paralysis was not required. The patient was mechanically ventilated in the left decubitus position. No further opioids or blood transfusions were required for the patient during the remainder of the procedure. 

During the thoracotomy stage, we achieved a tidal volume of 6-9 mL/kg with an intermittent peak pressure of 21 cmH2O and a positive end-expiratory pressure of 5 cmH2O and no interruptions in the surgery. An initial arterial blood gas analysis demonstrated a pH of 7.31 and a partial pressure of carbon dioxide of 48 mmHg with normal values in two subsequent samples. The ligation of TEF was completed uneventfully. The procedure was well tolerated by the patient, allowing the surgical team to proceed to a full repair of EA. 

Following the completion of the surgical repair, spontaneous ventilation was re-established. From the aortic cannula toiletry in the trachea, we removed a significant amount of bile, blood, and secretion, which had accumulated during the surgery, using a 6-FR soft-suction catheter. Subsequently, the aortic cannula was removed, and rigid bronchoscopy was performed for inspection and tracheal toiletry (Video 1). Although the otolaryngology team voiced concerns regarding the inflamed appearance of the subglottic section of the airway, the stenotic tracheal segment appeared normal on rigid bronchoscopy. A 3.5-mm uncuffed ETT was placed and positioned above the repaired TEF section using flexible bronchoscopy, with a review of the stenotic segment. The patient was transferred to the ICU intubated and sedated, with a propofol infusion of 175 µg/kg/min, and with adequate spontaneous ventilation. The cardiac surgery team was on standby for the initiation of ECMO in the event of respiratory failure at the end of the procedure or in the ICU. In the ICU, propofol was switched to morphine and dexmedetomidine infusions for sedation. Dexmedetomidine was weaned-off overnight to prepare for extubation the following day. 

The patient had an uneventful overnight course and was extubated on postoperative day (POD) 1 in the NICU by the same anesthesia team. From POD 2 to POD 5, the patient required a high-flow nasal cannula oxygen therapy with a peak of 8 L/min and a fraction of inspired oxygen of 40%, which were weaned down to 4 L/min and 21%, respectively. The patient was discharged from the NICU on POD 8 and is under long-term follow-up by the otolaryngology and general surgery teams. 

## Discussion

We present a rare case of TEF/EA associated with a severely long segment complete rings of the trachea with stenosis. The airway management was particularly challenging because of the severity of the tracheal stenosis and the necessary urgent surgery in the area of the defect. Congenital tracheal stenosis is typically caused by complete tracheal rings and is rarely associated with TEF/EA. Previous case reports have described the use of slide tracheoplasty to manage this issue surgically [1, 3-4]. We described the use of an aortic cannula to provide ventilation in the present case. 

The crucial first step in this patient’s care was the importance of multi-disciplinary discussions on the management of the anticipated difficult airway by providers with different expertise [4-6]. Benefits included shared mental models, consensus on an ideal algorithm for managing the patient, and assembly of various equipments, which might assist the medical and technical aspects of the patient’s care throughout the perioperative period. 

The second important factor was the concept of “institutional memory.” In our situation, the availability of one of the co-authors of the case published 13 years ago expedited the process of identifying a device suitable in the management of this case. While case reports are considered a low level of evidence for interventions, they have demonstrated utility in reporting novel techniques, which often lead to larger-scale trials [6-7]. Following this case, we performed a literature search of the keywords “difficult intubation” and “difficult airway” limited to case reports published from 1980 to 2018. After the removal of the duplicates and screening of abstracts, we identified more than 2000 airway-related case reports. This is a staggering number of case reports on airway management, with many of them behind pay-walls, potentially limiting their access. The creation of searchable databases of these published cases might assist clinicians in identifying novel techniques that have succeeded elsewhere. 

Advancements in diagnostic imaging made it possible for the anesthesia team to perform 3D reconstruction of the patient’s airway [8-10]. This allowed the anesthesia team to view, plan, prepare, and simulate the use of a nontraditional airway device. 

## Conclusions

In summary, this is a case report of TEF/EA and tracheal stenosis requiring the use of an aortic cannula for mechanical ventilation. This report emphasized the importance of developing innovative management strategies in clinically rare and challenging situations, the value of institutional history and publication of case reports, the significance of a multi-disciplinary approach, and the vital role that technological advancements play in the preparation for difficult cases. 
